# In Vitro Characterizations of Post-Crosslinked Gelatin-Based Microspheres Modified by Phosphatidylcholine or Diammonium Phosphate as Antibiotic Delivery Vehicles

**DOI:** 10.3390/polym15061504

**Published:** 2023-03-17

**Authors:** Kai-Chi Chang, Pei-Jheng Chang, Jian-Chih Chen, Ssu-Meng Huang, Shih-Ming Liu, Chi-Jen Shih, Wen-Cheng Chen

**Affiliations:** 1 Advanced Medical Devices and Composites Laboratory, Department of Fiber and Composite Materials, Feng Chia University, Taichung 407, Taiwan; 2 Department of Orthopedics, Faculty of Medical School, College of Medicine, Kaohsiung Medical University, Kaohsiung 807, Taiwan; 3 Department of Orthopedics, Kaohsiung Municipal Siaogang Hospital, Kaohsiung 807, Taiwan; 4 Department of Fragrance and Cosmetic Science, College of Pharmacy, Kaohsiung Medical University, Kaohsiung 807, Taiwan; 5 Drug Development and Value Creation Research Center, Kaohsiung Medical University, Kaohsiung 807, Taiwan; 6 Department of Medical Research, Kaohsiung Medical University Hospital, Kaohsiung 807, Taiwan; 7 Dental Medical Devices and Materials Research Center, College of Dental Medicine, Kaohsiung Medical University, Kaohsiung 807, Taiwan

**Keywords:** hydrogel, microspheres, emulsification, modification, drug release, antibiotic, biocompatibility

## Abstract

Hydrogel-based microspheres prepared by emulsification have been widely used as drug carriers, but biocompatibility remains a challenging issue. In this study, gelatin was used as the water phase, paraffin oil was used as the oil phase, and Span 80 was used as the surfactant. Microspheres were prepared using a water-in-oil (W/O) emulsification. Diammonium phosphate (DAP) or phosphatidylcholine (PC) were further used to improve the biocompatibility of post-crosslinked gelatin microspheres. The biocompatibility of DAP-modified microspheres (0.5–10 wt.%) was better than that of PC (5 wt.%). The microspheres soaked in phosphate-buffered saline (PBS) lasted up to 26 days before fully degrading. Based on microscopic observation, the microspheres were all spherical and hollow inside. The particle size distribution ranged from 19 μm to 22 μm in diameter. The drug release analysis showed that the antibiotic gentamicin loaded on the microspheres was released in a large amount within 2 h of soaking in PBS. It was stabilized until the amount of microspheres integrated was significantly reduced after soaking for 16 days and then released again to form a two-stage drug release curve. In vitro experiments showed that DAP-modified microspheres at concentrations less than 5 wt.% had no cytotoxicity. Antibiotic-impregnated and DAP-modified microspheres had good antibacterial effects against *Staphylococcus aureus* and *Escherichia coli*, but these drug-impregnated groups hinder the biocompatibility of hydrogel microspheres. The developed drug carrier can be combined with other biomaterial matrices to form a composite for delivering drugs directly to the affected area in the future to achieve local therapeutic effects and improve the bioavailability of drugs.

## 1. Introduction

For centuries, routes of drug administration have generally been categorized according to the site of drug application, such as oral, intramuscular, and intravenous. Although these methods can play a therapeutic role in the affected area, they must be administered continuously to maintain the drug concentration, which not only depends on convenience but also on the properties and pharmacokinetics of the drug. The delivery of the drug can lead to the problem of overdosing, which increases the negative effects of the drug on the body. Therefore, some researchers began to study drug carriers for controlled drug release [1,2]. In this method, the drug can be released directly and locally through the drug carrier, which can control the drug release concentration and avoid the loss caused by a large number of passages and metabolism after delivery [3,4].

The use of drug carriers can overcome some problems related to the development and application of new drugs, but their use still has limitations, such as fast drug release, differences in solubility and biodistribution, etc., which will affect the interaction between the drug and the treatment site [5,6,7]. To optimize drug efficiency and reduce its side effects, many studies have prepared drug carriers by using natural or synthetic polymers through different techniques, such as emulsification, solvent evaporation, ionic gelation, self-assembly, nanoprecipitation, and supercritical fluid technology [8,9]. Emulsification is the most commonly used strategy to prepare polymer-based drug carriers. Related research and application of functional polymer colloids prepared by emulsification in biomedicine are increasing [10]. In an emulsion system, if the hydrophilic–lipophilic balance value of the surfactant is between 3.0 and 7.5, then it is suitable for water-in-oil (W/O) emulsion. A common surfactant is sorbitan oleate (span 80), which is an emulsifier/surfactant widely used in food and oral pharmaceuticals [11,12]. Paraffin oil is a type of mineral oil that is a by-product of the distillation of crude oil. Paraffin oil is often used as the oil phase of emulsions due to its good chemical stability and higher purity than other oils [13].

In this experiment, W/O emulsification was used to prepare micron-sized drug carriers, and gelatin was selected as the basic hydrogel for preparing microspheres. Gelatin is non-toxic, non-irritating, biodegradable, and easy to gel, resulting in a good molding effect; it has been widely used in the production of various drug delivery systems in recent years [14]. As a crosslinking agent, glutaraldehyde can quickly react with gelatin to form new covalent bonds and form a network connection structure [15]. However, microspheres made from materials, such as glutaraldehyde and paraffin oil, are cytotoxic during treatment, hampering their therapeutic efficacy [16,17]. In this regard, phosphatidylcholine (PC) and diammonium phosphate (DAP) were chosen to modify post-crosslinked microspheres. PC is a lipid mainly extracted from soybeans or egg yolks. It is commonly used in drug coatings and is a major component of the extracellular matrix [18], thus potentially providing the ability to modify the cellular affinity of microspheres. DAP is a water-soluble salt that is often used as a fertilizer to increase the pH of soil. Accordingly, microspheres can be modified after DAP is dissolved, mainly to adjust the pH value of the microspheres and improve cell compatibility [19].

In this study, W/O emulsification was used to prepare micron-sized spherical particles as drug carriers, which were modified with PC or DAP to improve their biocompatibility. Gentamicin was impregnated to observe its antibiotic release behavior and evaluate its bacteriostatic effect. The expected drug release effect of the modified microspheres was determined to evaluate the possible application of its combination with other biomaterial matrices in the affected area for topical treatment. In this regard, cytotoxicity was initially assessed in vitro.

## 2. Materials and Methods

### 2.1. Materials

Gelatin (type B from bovine hide, average molar mass 40,000–50,000 g/mole, Sigma-Aldrich^®^, St. Louis, MO, USA), parafilm oil, and phosphatidylcholine (PC) were purchased from Sigma-Aldrich Co. (St. Louis, MO, USA). Sorbitan monooleate-based Span 80 biodegradable nonionic surfactant was obtained from Nihon Shiyaku Reagent (Kyoto, Japan). Glutaraldehyde crosslinker, gentamicin antibiotic, and diammonium phosphate (DAP) were supplied by Panreac Química SLU (Barcelona, Spain), Zhaoguan Chemical Industry Co., Ltd. (Chiayi, Taiwan), and High Standard Enterprise Co., Ltd. (Taichung, Taiwan), respectively.

### 2.2. Microsphere Preparations

Gelatin-based microspheres were prepared by water-in-oil (W/O) emulsified solvent diffusion method with surfactant Span 80 as an auxiliary agent. About 1.5 g of gelatin powder was dissolved in 10 mL of deionized water under constant stirring at 50–60 °C to prepare 15% (*w*/*v*) gelatin solution as the water phase. Span 80 (0.2 mL) was dispersed into 20 mL of paraffin oil evenly and heated to 60 °C to form an oil phase.

About 3 mL of the gelatin solution was slowly dropped into the oil phase solution under constant stirring for 30 min to form emulsified droplets in the W/O emulsion. The emulsified droplets turned into gel particles during the cooling-down phase to 5 °C, which ensured that the emulsified droplets would not become a gelatin solution again. The gelatin microspheres were post-crosslinked by immersion into 1.5 mL of 15 wt.% glutaraldehyde for 30 min. Microspheres were collected by filtration, washed three times with acetone to remove excess oil phase substances, washed three times with deionized water, and then dried to obtain microspheres (Ms) before modification.

### 2.3. Microsphere Modification

#### 2.3.1. Solutions for Microsphere Modification

In this experiment, 5 wt.% PC and 1 wt.% DAP were used to modify the microspheres at room temperature. The microspheres were soaked in PC and DAP solutions for 24 h. The powder-to-solution ratio was 1/250 (g/mL), and PC-modified microspheres (denoted as pc-M) and DAP-modified microspheres (dap-M) were obtained after drying. The cytotoxicity of the modified microspheres was compared. The choice of modifying the microspheres with 0.5, 1, 2.5, 5, and 10 wt.% DAP concentrations was verified in detail, and expressed as 0.5, 1, 2.5, 5, and 10 dM groups, respectively. The cytotoxicity of the modified microspheres with different DAP concentrations was also compared.

#### 2.3.2. DAP-Modified Microspheres as Antibiotic Carriers

The antibiotic gentamicin was selected as the impregnated drug for release and antibacterial tests. Gentamicin at a stock concentration of 40 mg/mL was mixed into double-distilled water (ddH_2_O) to obtain 50 mL gentamicin (2 mg/mL) solution. The microspheres were immersed in the gentamicin solution at a liquid ratio of 1/50 (g/mL) for 24 h and freeze-dried to obtain microspheres loaded with gentamicin (0.5 dM-G, 1.0 dM-G, and 2.5 dM-G).

### 2.4. Characterization of Modified Microspheres

#### 2.4.1. Analysis of Infrared Spectroscopy and Morphologies

Fourier-transform infrared spectroscopy (FTIR; Nicolet 6700, Thermo Fisher Scientific, Waltham, MA, USA) was used to analyze changes in the functional groups of the gelatin microspheres after glutaraldehyde crosslinking and determine the crosslinking efficiency of the microspheres. Post-crosslinking and modified microsphere morphology were examined by scanning electron microscopy (SEM; Hitachi S-3400N, Hitachi, Tokyo, Japan).

#### 2.4.2. Crosslinking Index Changes

Ninhydrin (2,2-dihydroxy-1,3-indanedione, Sigma-Aldrich^®^, St. Louis, MO, USA) reacts with free amine groups to form a blue-purple product, with darker color indicating reaction. The presence of more amino groups leads to a lower degree of crosslinking. Residual amino groups of the microspheres before and after crosslinking were determined at an optical density wavelength (OD_570_) of 570 nm by using an enzyme-linked immunosorbent assay microplate (ELISA) reader (EZ Read 400, Biochrom, Cambridge, UK). Fixation index was calculated as follows [20]:

$$\mathrm{Fixation}\;\mathrm{index}\;\left(\%\right)=\frac{{\left(\mathrm{activated}\;\mathrm{amino}\right)}_{\mathrm{fresh}}-{\left(\mathrm{activated}\;\mathrm{amino}\right)}_{\mathrm{residual}}}{{\left(\mathrm{activated}\;\mathrm{amino}\right)}_{\mathrm{fresh}}}\times 100\;\%$$

The free amine group before crosslinking is (amine-reactive)_fresh_, and the free amine group after crosslinking is (amine-reactive)_residual_.

#### 2.4.3. Degradation of Microspheres In Vitro

The modified microspheres were immersed in deionized water and phosphate-buffered saline (PBS) for the degradation test. About 0.01 g of each group of microspheres was mixed with 1 mL of degradation solution into a microcentrifuge tube. Daily weight loss was recorded and converted to weight loss rate by using the following formula [21]:

$$\mathrm{Weight}\;\mathrm{loss}\;\left(\%\right)=\frac{W_{0}-W_{x}}{W_{0}}\times 100\;\%$$

where *W*_0_ is the weight of the microspheres accurately measured, and *W_x_* is the weight of the microspheres soaked in the degradation solution on the *x*-th day.

### 2.5. Antibiotic Release

Gentamicin released from the microspheres was measured using an ultraviolet/visible spectrophotometer (UV/Vis; UV1800, Shimadzu, Kyoto, Japan). The OD_202_ value of the gentamicin released from the microspheres in PBS was recorded within a certain period. Since gentamicin has the highest characteristic absorption at an optimal wavelength of 202 nm [22], a drug calibration curve was made and the absorptions at each time point were observed to determine the drug release profile.

### 2.6. Antibacterial Activity

Antimicrobial activity was measured by agar diffusion test using tryptic soy broth and agar (Neogen, Lansing, MI, USA). Anti-active bacteria were selected from *Staphylococcus aureus* and *Escherichia coli*. About 0.003 g of the sample powder was pasted on an agar culture plate and incubated at 37 °C for 24 h. The size of the inhibition zone was measured. The samples were cultured in the bacterial suspension for 1, 4, 8, 24, and 48 h to evaluate antibacterial growth ability [23]. About 100 μL of the suspension was collected to measure optical density with OD_595_ by ELISA microplate reader.

### 2.7. Cytotoxicity In Vitro

Fibroblast NIH-3T3 and L929 cells were used for cytotoxicity assays. Media and serum were purchased from Gibco^®^, Thermo Fisher Scientific Inc., Waltham, MA, USA. NIH-3T3 cells were cultured in Dulbecco’s modified Eagle medium (DMEM) containing 10% bovine serum. L929 cells were cultured in minimal essential medium alpha medium (MEMα) containing 10% horse serum.

NIH-3T3 and L9292 cells cultured in pure medium were set as control groups. The positive control was 15 vol.% dimethyl sulfoxide (DMSODMSO), and the negative control was high-density polyethylene (HDPE) extract to verify sample sterility. The microspheres were soaked in the medium at a ratio of 1:10 for 24 h as sample extracts. The cells were inoculated in a 96-well culture plate at a concentration of 1 × 10^4^ and cultured for 24 h. XTT reagent (Biological Industries, Kibbutz Beit Haemek, Israel; 50 μL/wells) was used to measure cell viability at OD_492_ by ELISA reader. Cell morphology was observed under an optical microscope (IVM-3AFL, SAGE VISION Co., Ltd., New Taipei City, Taiwan).

### 2.8. Statistical Analysis

Analysis of variance (ANOVA) and two-sample *t*-test in IBM SPSS Statistics Version 20 (IBM, New York, NY, USA) were used to evaluate differences between groups. Values at *p* < 0.05 were considered statistically significant.

## 3. Results and Discussion

### 3.1. Evaluation of Modified Microspheres by Cytotoxicity Assay

**Figure 1:**
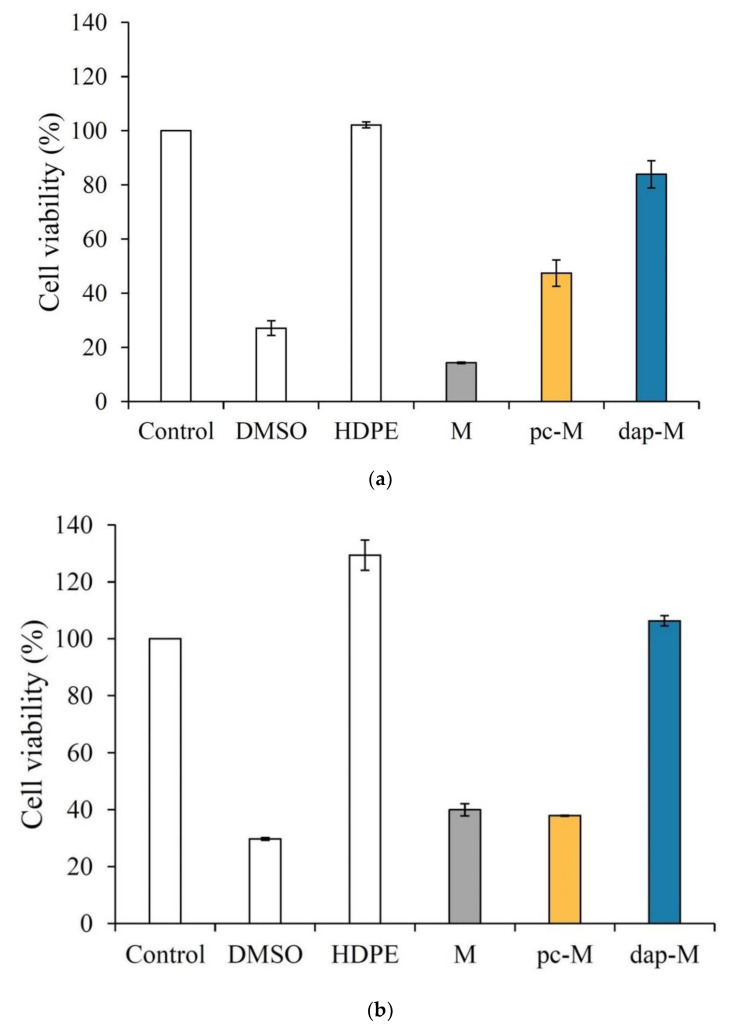

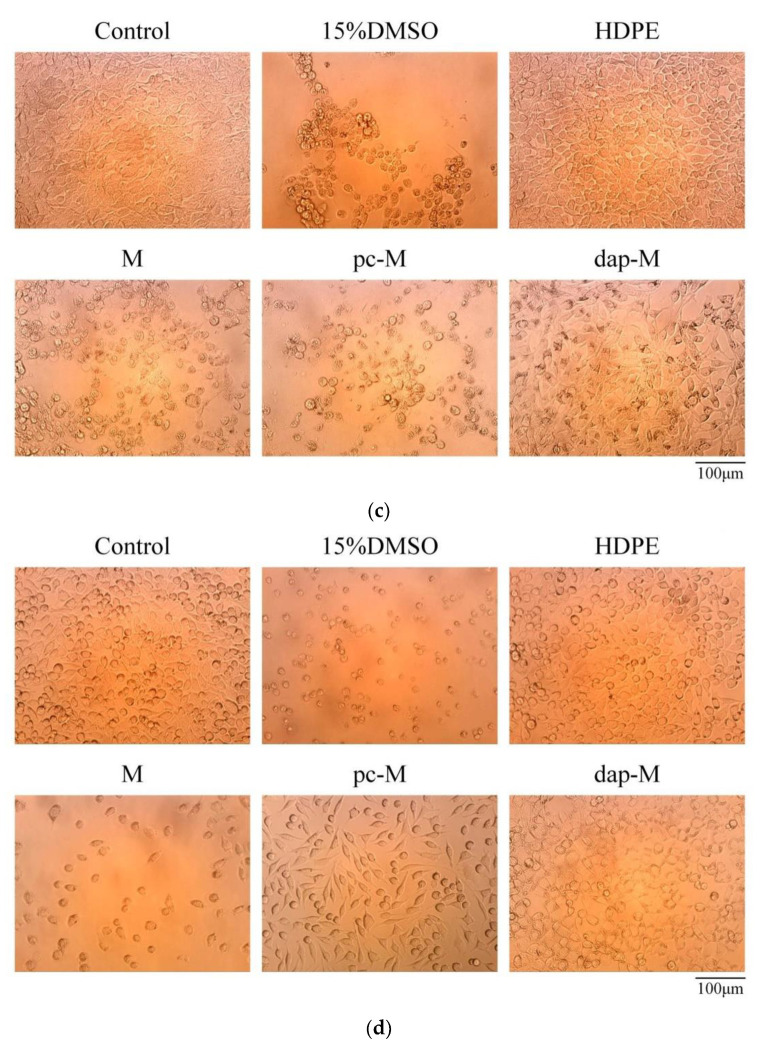
Cytotoxicity of gelatin-based microspheres modified with 5% PC (pc-M) and 1% DAP (dap-M): quantifications of (**a**) 3T3 and (**b**) L929 (*n* = 3) and qualitative observation of (**c**) 3T3 and (**d**) L929 cell lines.

According to the ISO 10993-5 regulation, cell viability maintained above 70% compared with the control group indicates no cytotoxicity, and values between 50–70% and 30–50% indicate slight and mild reactivity, respectively [24]. As shown in Figure 1a, the cell viability of the unmodified microspheres (Ms) against NIH-3T3 was only 14%, indicating that they had obvious cytotoxicity to the tested NIH-3T3 cells. The phosphatidylcholine-modified microspheres (pc-Ms) showed a cell survival rate of 47%, while the cell viability of DAP-modified microspheres (dap-Ms) improved, reaching 83% non-toxicity. The viability of L929 cells also showed the same trend (Figure 1b), and the value in the dap-M group reached as high as 106%, confirming that DAP instead of PC can greatly improve the biocompatibility of post-crosslinked microspheres. The cell morphology of the NIH-3T3 and L929 cultures in the pc-M group extracts was dead spheres, while the DAP-modified cells showed a good spindle shape, confirming that the cytotoxicity of the microspheres can be improved by modifying DAP (Figure 1c,d).

**Figure 2:**
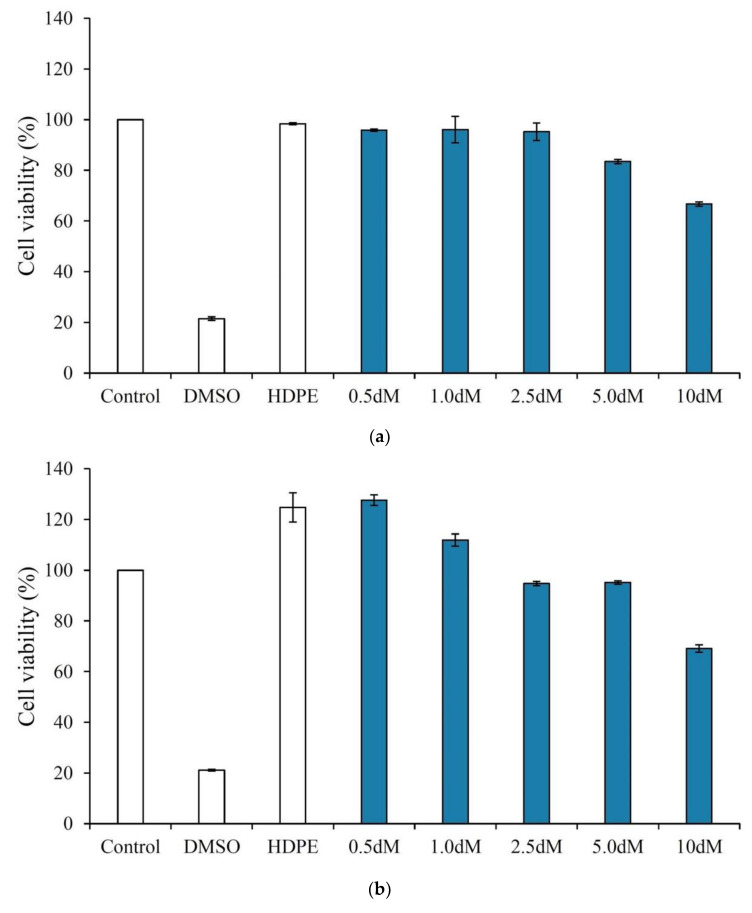

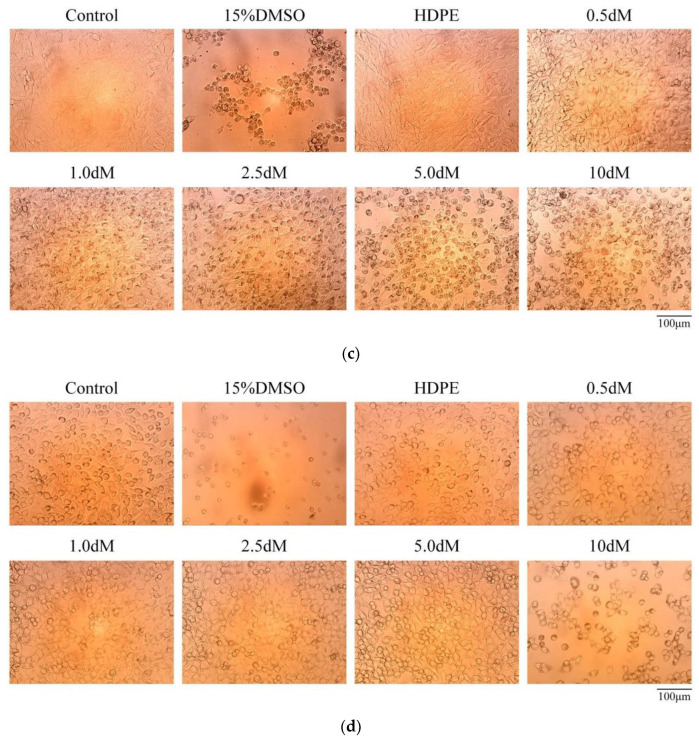
Cytotoxicity of modified microspheres with different concentrations of DAP (**a**) 3T3 and (**b**) L929 (*n* = 3) and qualitative observation of (**c**) 3T3 and (**d**) L929 cell lines.

Figure 2 shows the biocompatibility of the crosslinked microspheres modified with different concentrations of DAP. The viability of NIH-3T3 cells in the 0.5 dM, 1.0 dM, 2.5 dM, and 5.0 dM groups exceeded 70%, indicating that all the groups, except for the 10 dM group that had the highest DAP concentration, were not cytotoxic (Figure 2a). Similarly, L929 cells showed the same viability trend (Figure 2b). In the qualitative observation, cells in all groups, except for the 10 dM group, had a good spindle shape (Figure 2c,d).

The unmodified microspheres (Ms) had a pH of 7.02. The DAP-modified microspheres showed the same trend as DAP in the 0.5 dM, 1.0 dM, and 2.5 dM groups, which had pH of 7.66, 7.71, and 7.80, respectively, with increasing concentrations. Hence, the pH of the microspheres affects cytotoxicity.

In this experiment, the biomolecule PC was expected to be used as a surface modifier to increase the affinity of the microspheres to cells, but it was insufficient to attenuate the toxicity of the microspheres in NIH-3T3 and L929 cells. This finding may be caused by the acid stimulation of paraffin oil through the oil phase of the microsphere emulsification preparation [25,26]. However, basic DAP used for microsphere modification attenuated paraffin-stimulated cytotoxicity. In addition, the alkaline environment provided by DPA induced Span 80 to undergo saponification with paraffin oil to achieve the washing effect in the oil phase [27,28,29]. The resulting DAP-modified microspheres possess good compatibility without adverse effects on cells. To ensure the safety of DAP-modified microspheres, this study selected 0.5 dM, 1.0 dM, and 2.5 dM groups for subsequent experiments.

### 3.2. Characterization of DAP-Modified Microspheres

#### FTIR Analysis

**Figure 3:**
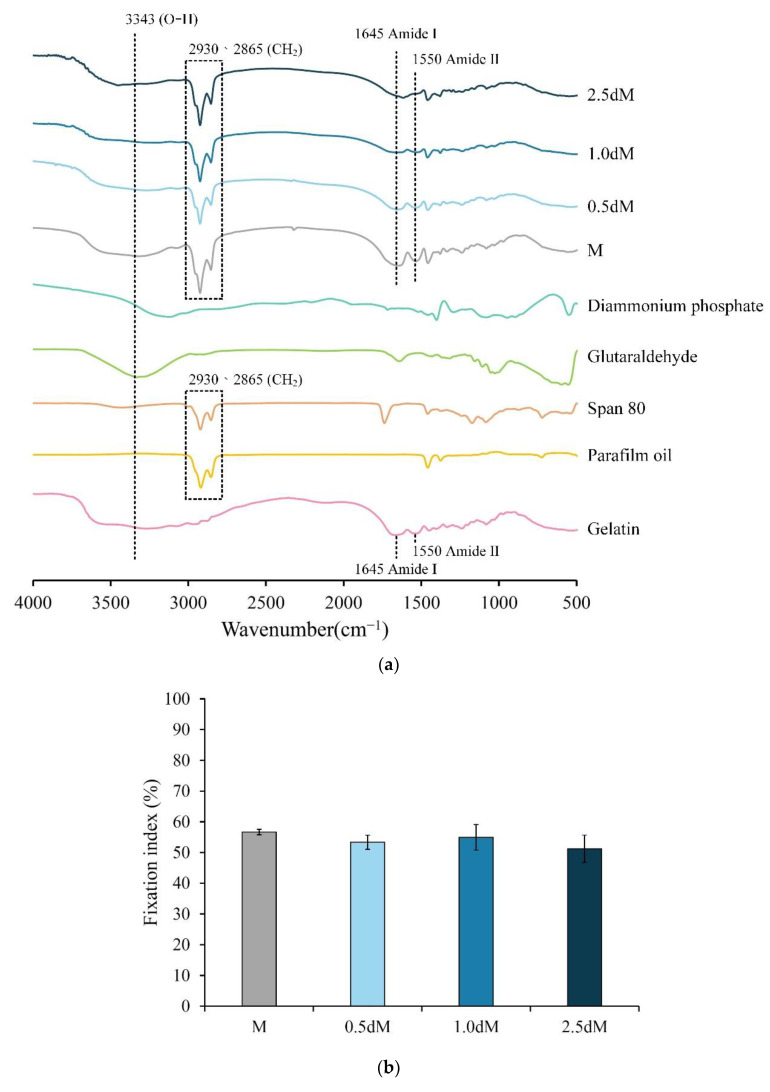

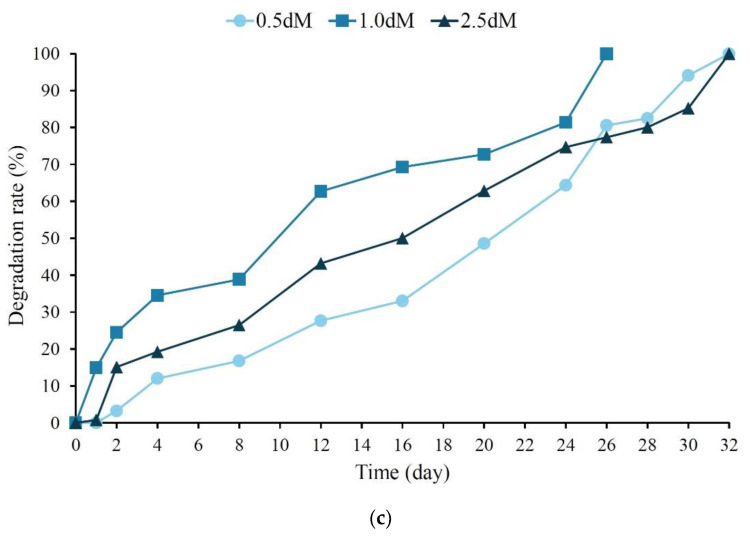
(**a**) FTIR spectra of raw materials, crosslinker, and microspheres modified with different concentrations of DAP. (**b**) Fixation of amine groups on microspheres modified with different concentrations of DAP to evaluate the degree of crosslinking (*n* = 3, *p* > 0.05). (**c**) Weight loss of DAP-modified microspheres with different concentrations soaked in PBS for 32 days (*n* = 3).

As shown in Figure 3a, gelatin has the absorption bands of amide I and amide II at 1550 and 1645 cm^−1^, respectively. Amide I is the stretching vibrations of C=O and C-N, and amide II is mainly due to the stretching vibrations of C-C and C-N [30,31,32]. Groups M, 0.5 dM, 1 dM, and 2.5 dM have gelatin absorptions of amide I and amide II. Functional groups, including paraffin oil and Span 80, were found in the oil phase residues because microspheres were prepared from W/O emulsions. Therefore, the prepared microspheres have C-H_2_ absorption at 2865 and 2930 cm^−1^ bands. In addition, the microspheres and crosslinker glutaraldehyde have an O-H absorption peak at the 3343 cm^−1^ band. Although gelatin also has O-H absorption, the microspheres could be free of glutaraldehyde residues because of a lack of tendency for an increase in O-H density.

### 3.3. Fixation of Free Amine

The amine fixation index indicates that the crosslinking reaction was carried out by glutaraldehyde. The degree of crosslinking was approximately between 51% of the 2.5 dM group and 56% of the M group (Figure 3b). The difference in amine immobilization was not statistically significant (*p* > 0.05), indicating that DAP modification did not significantly affect the crosslinking of microspheres.

### 3.4. Degradation of DAP-Modified Microspheres

As shown in Figure 3c, 0.5 dM and 2.5 dM were completely degraded within 32 days, while 1.0 dM was degraded within 26 days. During this period, each group of microspheres did not degrade suddenly and rapidly but degraded in a linear manner: 0.5 dM (y = 2.9807x − 4.5438, R^2^ = 0.9735), 1.0 dM (y = 3.1905x + 13.844, R^2^ = 0.9434), and 2.5 dM (y = 2.9257x + 3.8528, R^2^ = 0.9865). From the perspective of linear relationships, no significant difference was found among the three groups. However, the initial minimum degradation rate was 0.5 dM, indicating that the modified microspheres of 0.5 dM exhibited the best resistance to degradation.

### 3.5. Observation of Gelatin-Based Microspheres via DAP Modification

**Figure 4:**
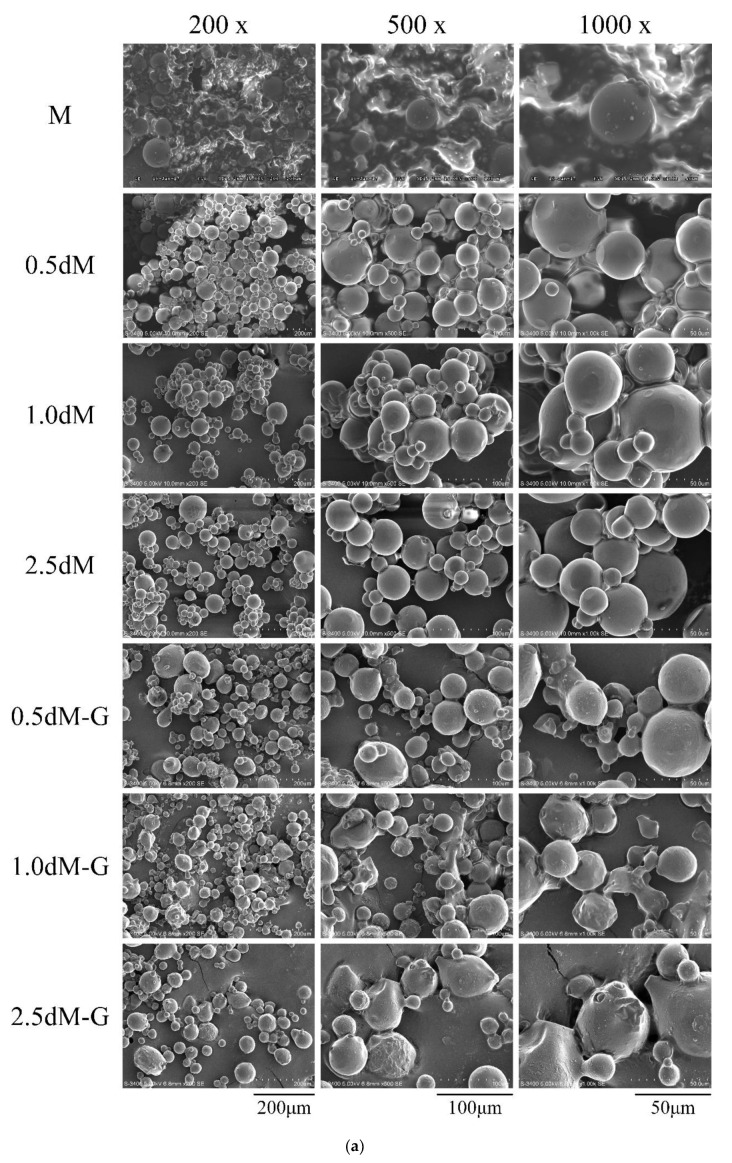

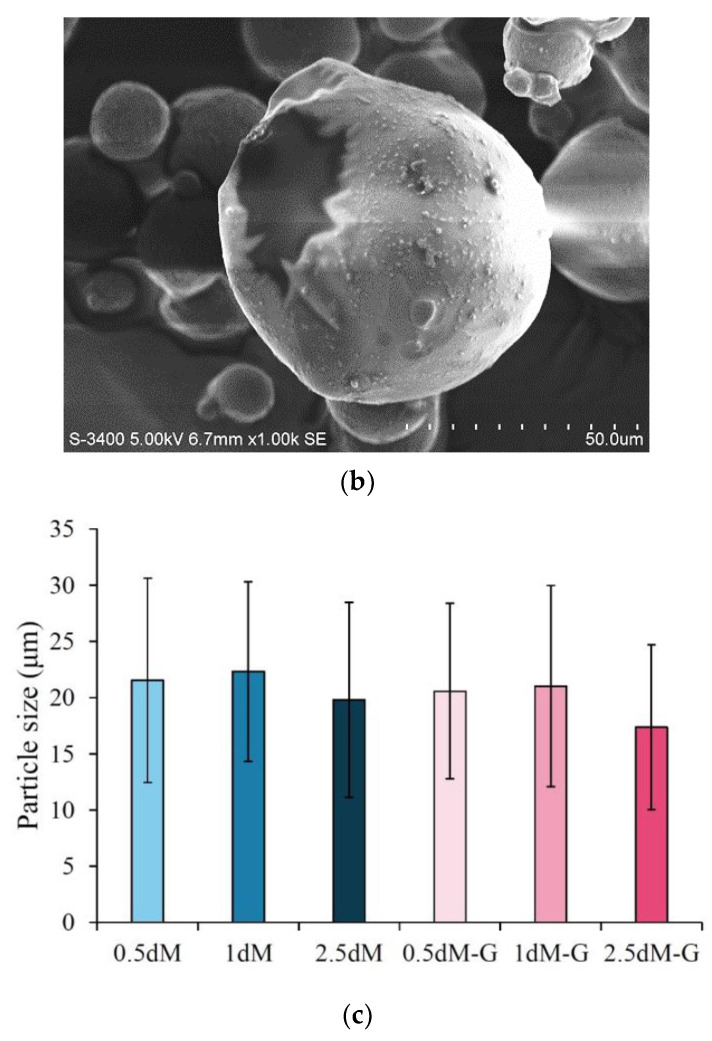
Morphology of unmodified microspheres and microspheres modified with different concentrations of DAP and PC: (**a**) SEM image, (**b**) microspheres with cracks observed to further confirm that the interior is hollow, and (**c**) microsphere diameter measurement (*n* = 100, *p* > 0.05).

Based on the observation of the microstructure of the unmodified microsphere group M (Figure 4a), gelatin emulsification formed spherical microspheres, which have smooth surfaces without pits and are fine-grained. However, the oil phase dispersion was observed among the gelatin-based microspheres because microspheres were prepared by W/O emulsification. After modifying the microsphere groups with different DAP concentrations of 0.5 dM, 1.0 dM, and 2.5 dM, the microspheres in each group were still spherical and the surface was smooth. After modification, oil phase dispersion was not found around the microspheres. Several groups of microspheres impregnated with gentamicin were oval-shaped, and a few microspheres had wrinkled surfaces. The microspheres may be deformed due to tension changes caused by ice sublimation during the use of freeze-drying to dehydrate under low pressure; most of the microspheres remained spherical after freeze-drying [33]. The ruptured microspheres are presented in Figure 4b. The interior of the hydrogel microspheres was hollow, except for the drug attached on the outside of the microspheres, thereby increasing the drug loading inside and controlling the release effect of the microspheres [34].

The average size of the modified microspheres was calculated by SEM analysis (Figure 4c; *n* = 100). The particle sizes of 0.5 dM-, 1.0 dM-, and 2.5 dM-modified microspheres were 21.54 ± 9.09, 22.32 ± 7.97, and 19.81 ± 8.67 μm, respectively; the particle sizes of gentamicin microspheres impregnated with 0.5 dM-G, 1.0 dM-G, and 2.5 dM-G decreased slightly, with values of 20.56 ± 7.81, 21.09 ± 8.95, and 17.36 ± 7.33 μm, respectively. Statistical analysis was carried out in each group, and no significant difference was found (*p* > 0.05).

The modified hydrogel microspheres have the advantages of simple preparation process, uniform particle size, large specific surface area, long degradation time, and slow drug release. Microspheres in bioinks or hydrogels are used as injectable biomaterials for tissue engineering, such as in hard bone, cartilage, and liver. The particle size should be in an appropriate range of 20–200 μm [35,36,37]. Figure 4 shows that microspheres with uniform shape and small particle size (~20 μm) were successfully prepared by simple W/O emulsification. By contrast, conventional needle with 18-gauge injection had difficulty delivering particles with diameters greater than 200 μm. Hence, microspheres prepared in this study are suitable for co-injection with other biological fluids. Microspheres with tailored porosity exhibit greater surface area, lower mass density, and better attachment and cellular interactions for cell proliferation, drug absorption, and drug release kinetics compared with macrospheres [38]. Dong et al. [39] showed that gelatin-based microspheres have great potential as cell carriers and 3D scaffold components in tissue engineering and regenerative medicine because they not only have excellent injectability but can also be integrated with large-scale structures loaded with cells.

### 3.6. Antibiotic Release

**Figure 5:**
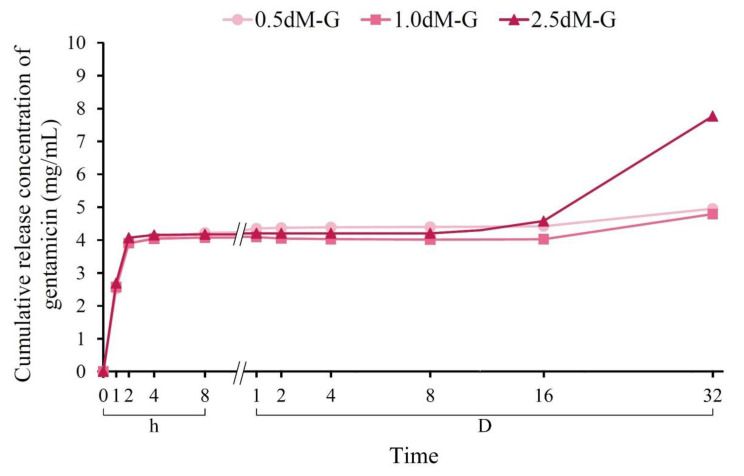
Antibiotic release of DAP-modified microspheres impregnated with antibiotic gentamicin (*n* = 6).

From the release curve of gentamicin (Figure 5), the release amount in the three groups of drugs in the first 2 h was about 4 mg/mL, which was mainly via surface diffusion release, and the release amount remained unchanged. The second drug release was not evident until 16 days after soaking. On the 32nd day, the drug release amounts of 0.5 dM-G, 1.0 dM-G, and 2.5 dM-G reached 4.9, 4.7, and 7.7 mg/mL, respectively. When each group of microspheres began to degrade and the hydrogel underwent structural slippage, the drug encapsulated in the microspheres was released, resulting in two-stage drug release behavior [40].

The cumulative antibiotic release of 2.5 dM-G was not significantly higher than that of 0.5 dM-G and 1.0 dM-G until the microspheres showed a significant difference after soaking for 32 days. The release was not significant for a period of time, but the cumulative antibiotic dose was effective for 4–8 h of antibacterial ability, and the release amount maintained a plateau for 16 days. Compared with the degradation of DAP-modified microspheres after soaking, the post-crosslinked hydrogel resulted in less than half of the degradation of the dense network structure (Figure 3c), thereby efficiently decreasing the swelling rate and the drug release rate of the hydrogel microsphere matrix [41].

Prolonged drug release was observed in this study, possibly because of freer void spaces available, through which fewer drug molecules will be transported. The cumulative release percentage of 2.5 dM-G was higher than that of 0.5 dM-G and 1.0 dM-G. The increase in DCP in the hydrogel matrix helps to carry large doses of antibiotics, resulting in higher release from gentamicin in the matrix. This secondary release explains that the explicit release behavior of post-crosslinked hydrogel microspheres depends not only on many factors, such as degree of crosslinking, but also drug loading amount and method [42].

### 3.7. Antibacterial Abilities

**Figure 6:**
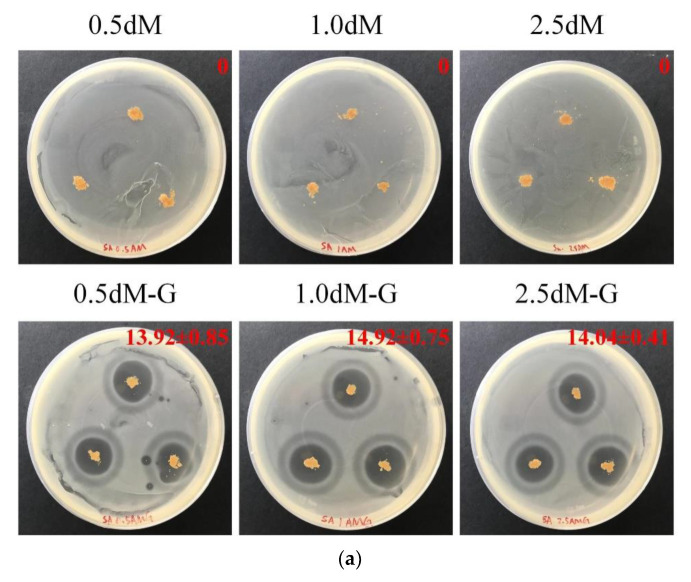

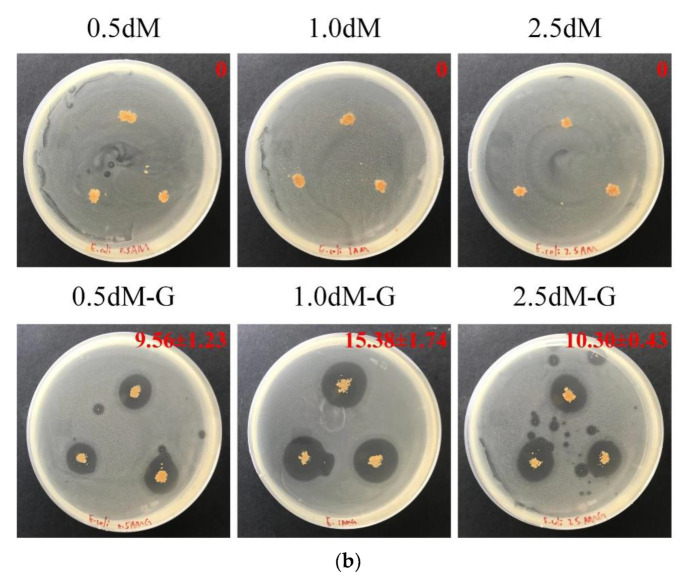
Antibacterial activities of antibiotic-free and DAP-modified microspheres impregnated with antibiotics against (**a**) *S. aureus* and (**b**) *E. coli* for a zone of inhibition of 1 day (*n* = 3).

Figure 6 shows the qualitative antibacterial results of sample groups against *S. aureus* and *E.coli*. The groups not impregnated with gentamicin had no zone of inhibition. However, the gentamicin-impregnated groups had obvious antibacterial zone formation, indicating a good antibacterial effect.

**Figure 7:**
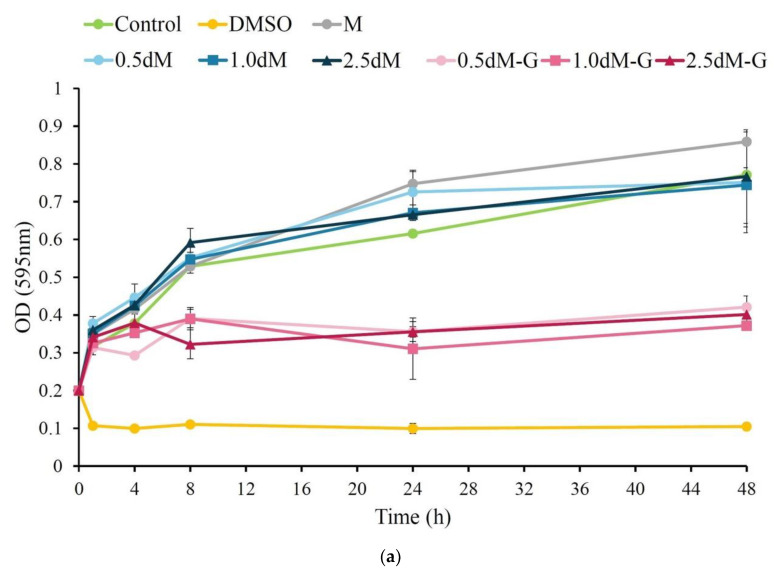

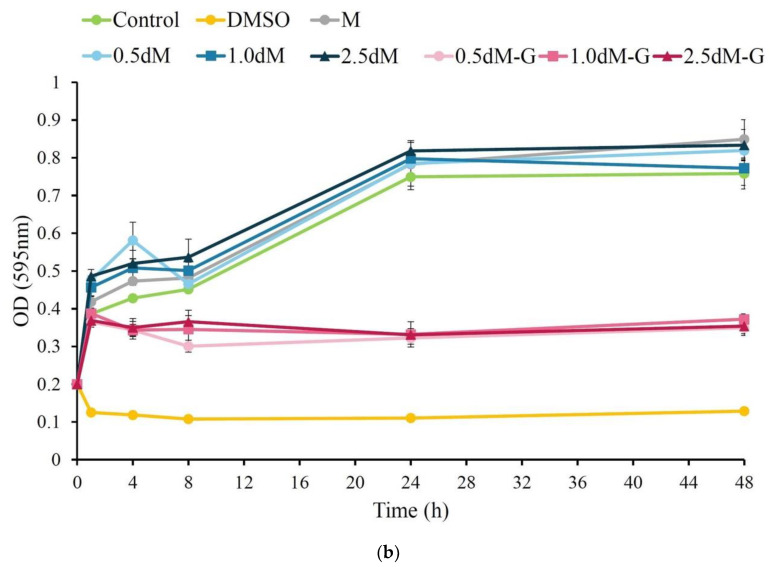
Quantitative analysis: antibacterial activities of antibiotic-free and DAP-modified microspheres impregnated with antibiotics against (**a**) *S. aureus* and (**b**) *E. coli* for 48 h (*n* = 3).

The microspheres in each group were soaked in *S. aureus* and *E. coli* suspensions for 1, 4, 8, 24, and 48 h. Based on the comparison of *S. aureus* and *E. coli*, the antibacterial activity was lower in 0.5 dM-G, 1.0 dM-G, and 2.5 dM-G than in the non-impregnated gentamicin group (Figure 7). The gentamicin group had obvious antibacterial ability after soaking for 4–8 h, indicating that the antibiotic-impregnated microspheres successfully released the drug to achieve the antibacterial effect.

The total degradation time of the emulsified microspheres prepared in this study was longer (about 30 days) compared with those of other hydrocolloid microspheres (Figure 3c and Figure 5). The drug release in the first 1 to 2 h of immersion was mainly due to the rapid release of the drug on the surface of the adsorbed microspheres. The drug was slowly diffused and released due to the swelling of the microspheres until 16 days. The structure of the microspheres was then greatly disassembled, causing the drug to undergo secondary release as the hydrogel structure slips [43,44]. Microspheres with sustained release capability are especially suitable for drug delivery in long-term clinical treatment operations to control the release of locally delivered drugs and improve the treatment efficiency [45]. For example, cartilage regeneration requires more than 30 days of growth factor release to achieve repair [46].

### 3.8. Biocompatibility of Microspheres Impregnated with Antibiotic

**Figure 8:**
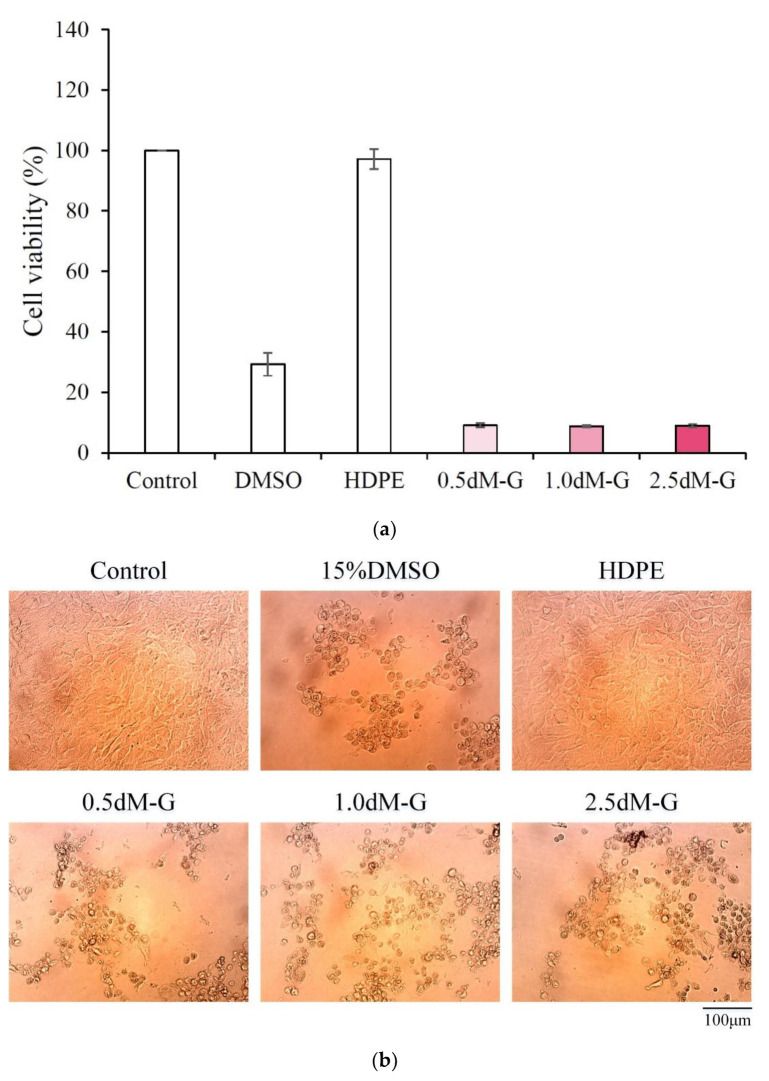
Extract culture with NIH 3T3 cells for 1 day; (**a**) quantitative (*n* = 3) and (**b**) qualitative measurements of antibiotic-impregnated microspheres.

As shown in Figure 8a, the cell viability of each group of microspheres was lower than 20%, showing obvious cytotoxicity. This finding may be due to the sudden release of excess gentamicin from the microspheres during the preparation of the extract, thereby reducing the cell viability. Based on the analysis of the morphology of 3T3 cells (Figure 8b), the extracts of microspheres impregnated with gentamicin caused apoptosis, leading to spherical and ruptured cells. Thus, the release of gentamicin will be toxic to NIH3T3 cells. In this study, DAP modification enhanced the cytotoxicity of the microspheres. However, the biocompatibility of the microspheres deteriorated after drug impregnation. Micro-hydrogels have certain limitations in repairing large-area wound defects so they cannot form accurate shapes to repair damage due to flow under blood infiltration [21,22,23,24,47]. Therefore, drug-impregnated microspheres as drug carriers can be composited into other biomaterials, such as artificial skin for burns or fractures, membranes for protein capture and wound healing, bone cement or polymethylmethacrylate for bone defect repair, and bioink for tissue engineering applications [48,49,50].

## 4. Conclusions

In this experiment, post-crosslinked gelatin-based microspheres were successfully prepared as drug carriers by W/O emulsification. Basic DAP modification could effectively improve the cytotoxicity of the microspheres and confer them with good biocompatibility. The surface of the microspheres was smooth, spherical in shape, and hollow inside. The size of the microspheres impregnated with gentamicin was not different from that without antibiotics. In terms of weight loss, 0.5 dM and 2.5 dM were completely degraded in 32 days and 1.0 dM was degraded in 26 days, indicating that each group of microspheres has the potential to be applied to long-acting drug delivery systems. From the drug release profile of gentamicin, the release curves of the 0.5 dM-G, 1 dM-G, and 2.5 dM-G groups were approximately the same. The release busted in the first 2 h; then, the release was stopped, and the amount reached a plateau until the microspheres were soaked for 16 days. Given the obvious degradation of the microspheres, the drug was slowly released again after soaking for 16–32 days, and this phenomenon is called secondary release. The gentamicin impregnation group of DAP-modified microspheres all achieved an antibacterial effect, which proved that the microspheres were successfully impregnated with drugs and released drugs to achieve the antibacterial effect. However, the biocompatibility of the drug-containing microspheres was not good. In summary, the gelatin microspheres prepared by W/O emulsification not only have good biocompatibility but also have the ability of drug loading and secondary release after modification with DAP. In the future, these microspheres are expected to be combined with other biomaterials as a carrier of drugs or growth factors for clinical applications.

## Data Availability

The data presented in this study are available on request from the corresponding author.
